# Heart rate/temperature ratio: A practical prognostic indicator for critically ill patients with sepsis

**DOI:** 10.1016/j.heliyon.2024.e24422

**Published:** 2024-01-11

**Authors:** Zongbin Lin, Shan Lin

**Affiliations:** a Department of Respiratory and Critical Care Medicine, Affiliated Hospital of North Sichuan Medical College, Nanchong, Sichuan, China; b Emergency and Critical Care Center, Intensive Care Unit, Zhejiang Provincial People's Hospital (Affiliated People's Hospital, Hangzhou Medical College), Hangzhou, Zhejiang, China

**Keywords:** Sepsis, Prognosis, Heart rate, Temperature

## Abstract

**Background:**

We hypothesize that the heart rate/temperature ratio can predict intensive care unit (ICU) mortality in critical ill patients with sepsis. We aimed to explore the association between the heart rate/temperature ratio and ICU mortality in patients with sepsis.

**Methods:**

We conducted this study utilizing a comprehensive critical care medicine database. The primary endpoint assessed was ICU mortality. A multivariable logistic regression model was employed to determine the independent impact of the heart rate to temperature ratio on ICU mortality.

**Results:**

The study included 12,321 patients. A nonlinear relationship was observed between the heart rate/temperature ratio and ICU mortality, with an inflection point identified at 2.22. The results from the Multivariable logistic regression analysis revealed that the heart rate/temperature ratio independently contributed to the risk of ICU mortality. In model II, there was a 55 % higher ICU mortality rate with a heart rate/temperature ratio greater than 2.22 than with that less than 2.22 (odds ratio [OR] = 1.55, 95 % confidence interval [CI] 1.35–1.77). Moreover, an elevated heart rate/temperature ratio as a continuous variable showed a positive association with ICU mortality (OR = 2.14; 95 % CI: 1.87–2.45). The impact of the heart rate/temperature ratio on ICU mortality remained consistent across all subgroup variables. The sensitivity analysis results consistently supported the primary outcome, with an E value of 2.47. This suggests that the influence of unmeasured confounders on the observed outcomes was minimal, thereby confirming the robustness of the findings.

**Conclusions:**

The heart rate/temperature ratio is a readily available and convenient indicator in a clinical setting. Elevated heart rate/temperature ratios, particularly those exceeding 2.22, are strongly linked to a high ICU mortality rate among critically ill sepsis patients.

## 1 Introduction

Activation of the sympathetic nervous system and the release of catecholamines are crucial events that occur after the onset of sepsis [1]. Initially, it was observed that epinephrine and norepinephrine inhibit histamine secretion in mast cells, suggesting an interaction between the sympathetic nervous system and the immune system [2,3]. Additionally, the intensity and duration of catecholamine release, which occurs in response to infection, can potentially affect the acute inflammatory response and activation of the immune system [4]. Sepsis is a complex organismal response to infection, and its pathophysiology involves a complex interaction between pro- and anti-inflammatory responses and the release of significant amounts of endogenous catecholamines and cytokines. This is evidenced by elevated body temperature and heart rate observed in patients [5]. In sepsis, the relationship between hypothermia and unfavorable outcomes has been well documented, with mortality decreasing as body temperature increases [6,7]. Similarly, several studies have identified tachycardia and heart rate variability as predictive factors of prognosis in both adult and pediatric sepsis [[8], [9], [10]]. Furthermore, one study concluded that patients with sepsis and persistent tachycardia, even after receiving adequate fluid resuscitation, had a worse prognosis [11]. This may have been due to excessive adrenergic stimulation and autonomic dysfunction. Significantly better outcomes were observed when heart rate control was achieved through the use of β-adrenergic blockers. However, the effect of β-blocker therapy on the clinical outcomes of sepsis remains unclear. Previous studies have explored the potential benefits of ultrashort blockers such as esmolol and landiolol in alleviating the sympathetic stress response in sepsis [12,13]. Additionally, a recent study revealed that long-acting β-blocker therapy may have a protective effect in patients with sepsis [14].

Heart rate and body temperature are commonly used clinical indicators. However, these indicators are affected by various contributing factors that limit their predictive value for disease. In contrast, the relative heart rate index, which is calculated by dividing the heart rate by the body temperature, eliminates the influence of body temperature on the heart rate. Our review of previously published studies indicates that only a small number of studies have investigated the role of the heart rate/temperature ratio in sepsis. The studies conducted by Leibovici et al. and Guz et al. revealed that a higher heart rate/temperature ratio was significantly associated with poor prognosis [15,16]. However, these findings were restricted by the small sample size and diagnosis of systemic inflammatory response syndrome (SIRS). It is crucial to investigate the prognostic value of the heart rate/temperature ratio in patients with sepsis on a larger scale. Hence, our objective was to examine the relationship between the heart rate/temperature ratio at intensive care unit (ICU) admission and mortality rate in patients with sepsis.

## 2 Methods

### 2.1 Patient data

This study utilized a large critical care medicine database, Medical Information Mart Intensive Care III (MIMIC-III) [17]. Access to the database was granted by the institutional review boards of 10.13039/100005615Beth Israel Deaconess Medical Center and Massachusetts Institute of Technology Affiliates (Record ID: 49780033). Patient consent was not required as the data were anonymized.

For this study, we included adult patients (age ≥18 years) with sepsis based on the criteria for sepsis 3.0. Patients with less than one day of ICU stay were excluded [5]. We did not exclude comorbidities that could potentially affect heart rate and temperature, considering their relevance in clinical practice. Most patients with sepsis requiring ICU admission have certain comorbidities and excluding them may limit the applicability of the heart rate/temperature ratio in this context. The codes that assisted MIMIC-III for data extraction are consistent with those in our previous studies and are publicly available on the website (https://github.com/MIT-LCP/mimic-code) [[18], [19], [20], [21]].

## 3 Outcomes

### 3.1 The primary outcome was ICU mortality

#### 3.1.1 Statistical analysis

For continuous variables, data are presented as mean ± standard deviation or median (interquartile range, IQR), and for categorical variables, data are presented as numbers and percentages. We compared the clinical characteristics between the two groups according to ICU outcomes (chi-square test for categorical variables and Student's t-test or Wilcoxon rank-sum test for continuous variables). First, the generalized additive model was used to explore whether there was a nonlinear relationship between the heart rate/temperature ratio and ICU mortality; if so, the log-likelihood ratio test was used to compare the two linear regression models, and then the inflection point was calculated by threshold effect analysis. Subsequently, the inflection point was used to categorize the heart rate/temperature ratio into two groups (<2.22, ≥2.22), and a multivariate logistic regression model was used to explore the independent effect of heart rate/temperature ratio on ICU mortality. Covariate selection was performed if a covariate changed the estimate of heart rate/temperature ratio on ICU mortality by >10 % or was markedly associated with ICU mortality. Next, interaction tests and stratified analyses were used to explore whether the heart rate/temperature ratio remained consistent across subgroup variables for outcomes. The difference in survival between the two groups in terms of the heart rate/temperature ratio (<2.22, ≥2.22) at 28 days was visualized using Kaplan-Meier analysis. All data were analyzed using EmpowerStats (www.empowerstats.com) and R (http://www. R-project. org/). *P*-values of <0.05 were considered to be statistically significant.

### 3.2 Sensitivity analysis

We conducted a sensitivity analysis to account for the potential influence of comorbidities, such as heart failure, arrhythmia, cardiac valve disease, and hypothyroidism, on the heart rate/temperature ratio. We performed multivariate logistic regression and smoothed curve fitting analysis after excluding these comorbidities. Additionally, we calculated E values to assess the potential impact of unmeasured confounders on the relationship between heart rate/temperature ratio and ICU mortality [22,23]. The E value was used to determine the magnitude of an unmeasured confounding factor required to negate the observed correlation between heart rate/temperature ratio and ICU mortality.

## 4 Results

### 4.1 Characteristics of the study population

A total of 12,321 patients with sepsis were included in this study. As shown in Table 1, there were 1309 non-survivors (10.62 %). In terms of basic characteristics, age, heart rate, heart rate/temperature ratio, Elixhauser Comorbidity index, SOFA score, and length of hospital stay were significantly higher in non-survivors than in survivors; however, the temperature was notably higher in survivors compared to non-survivors. There were no significant differences in the population receiving mechanical ventilation or renal replacement therapy between the two groups. Details of the remaining comorbidities are presented in Table 1.

**Table 1:** Characteristics of participants.

Variables	All patients (N = 12,321)	Survivors (N = 11,012)	Non-survivors (N = 1309)	*P-*value
Age (years)	67.10 ± 16.42	66.46 ± 16.53)	72.57 ± 14.28	<0.001
Sex				0.325
Male	6493 (52.70 %)	5820 (52.85 %)	673 (51.41 %)	
Female	5828 (47.30 %)	5192 (47.15 %)	636 (48.59 %)	
Heartrate (bpm)	88.08 ± 16.50	87.45 ± 16.09	93.31 ± 18.80	<0.001
Temperature (°C)	36.89 ± 0.69	36.91 ± 0.67	36.78 ± 0.87	<0.001
Heart rate/Temperature (bpm/°C)	2.39 ± 0.44	2.37 ± 0.43	2.54 ± 0.50	<0.001
Elixhauser Comorbidity index	17.00 (8.00–26.00)	16.00 (7.00–25.00)	22.00 (14.00–31.00)	<0.001
SOFA	5.00 (3.00–7.00)	5.00 (3.00–7.00)	6.00 (4.00–9.00)	<0.001
Length of ICU stay (days)	3.33 (1.83–7.86)	3.29 (1.81–7.87)	3.61 (1.94–7.83)	0.093
Length of hospital stay (days)	10.85 (6.36–19.04)	11.17 (6.71–19.73)	8.18 (4.70–14.70)	<0.001
Mechanical ventilation	6132 (49.77 %)	5483 (49.79 %)	649 (49.58 %)	0.885
Renal replacement therapy	604 (4.90 %)	531 (4.82 %)	73 (5.58 %)	0.232
Comorbidities, n (%)				
Congestive heart failure	4168 (33.83 %)	3666 (33.29 %)	502 (38.35 %)	<0.001
Cardiac arrhythmias	4173 (33.87 %)	3673 (33.35 %)	500 (38.20 %)	<0.001
Valvular disease	1747 (14.18 %)	1572 (14.28 %)	175 (13.37 %)	0.374
Peripheral vascular disease	1411 (11.45 %)	1246 (11.31 %)	165 (12.61 %)	0.166
Pulmonary circulation disease	985 (7.99 %)	862 (7.83 %)	123 (9.40 %)	0.048
Chronic pulmonary disease	2716 (22.04 %)	2418 (21.96 %)	298 (22.77 %)	0.505
Hypothyroidism	1364 (11.07 %)	1237 (11.23 %)	127 (9.70 %)	0.095
Hypertension	6522 (52.93 %)	5926 (53.81 %)	596 (45.53 %)	<0.001
Diabetes	3509 (28.48 %)	3181 (28.89 %)	328 (25.06 %)	0.004
Renal failure	2226 (18.07 %)	1990 (18.07 %)	236 (18.03 %)	0.970
Liver disease	1258 (10.21 %)	1029 (9.34 %)	229 (17.49 %)	<0.001
Tumor	1487 (12.07 %)	1238 (11.24 %)	249 (19.02 %)	<0.001

**Abbreviations:** ICU, intensive care unit; SOFA, sequential organ failure assessment.

### 4.2 Smooth curve fitting and threshold effect analysis

**Fig. 1:**
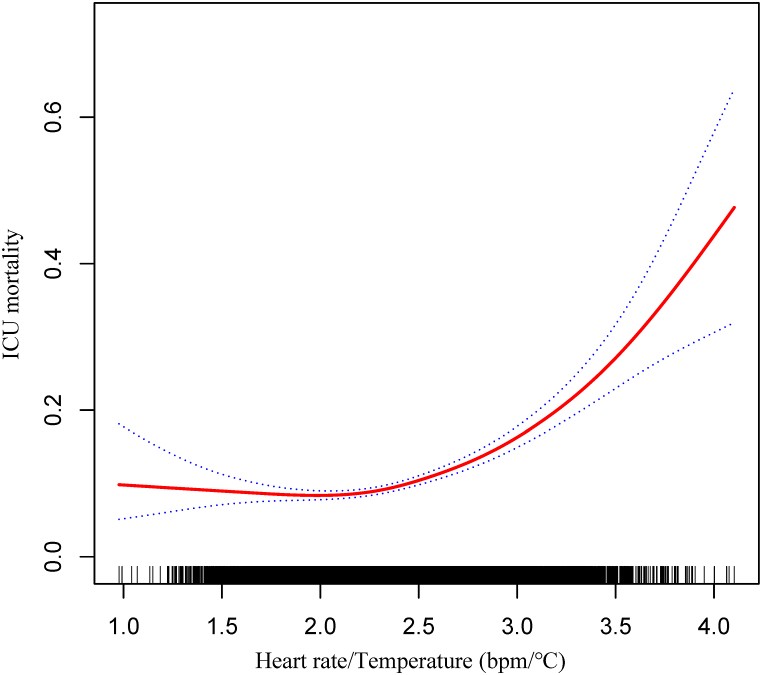
Association between heart rate/temperature ratio and ICU mortality. Note: adjusted by age, sex, SOFA, mechanical ventilation, renal replacement therapy, congestive heart failure, cardiac arrhythmias, valvular disease, peripheral vascular disease, pulmonary circulation disease, chronic pulmonary disease, hypothyroidism, hypertension, diabetes, renal failure, liver disease, tumor.

The two-piecewise linear regression model implied a nonlinear relationship between the heart rate/temperature ratio and ICU mortality (log-likelihood ratio test, *P* < 0.001 [Fig. 1 and Table 2]. Threshold effect analysis indicated an inflection point of 2.22, and when the heart rate/temperature ratio was less than 2.22, the effect of elevated heart rate/temperature ratio on ICU mortality was not remarkable (OR = 0.71, 95 % CI 0.47–1.06), whereas when the heart rate/temperature ratio was greater than 2.22, ICU mortality increased appreciably with the elevated heart rate/temperature ratio (OR = 3.00, 95 % CI 2.54–3.68) [Table 2].

**Table 2:** Threshold effect analysis of heart rate/temperature ratio and ICU mortality.

ICU mortality			
One-line linear regression model: OR = 2.14, 95 % CI (1.87–2.45), *P* < 0.0001			
The two-piecewise linear regression model			
Inflection point	OR	95 % CI	*P*-value
<2.22	0.71	0.47–1.06	0.0964
≥2.22	3.00	2.54–3.68	<0.0001
The log-likelihood ratio test: *P* < 0.001			

**Abbreviations:** ICU, intensive care unit; OR, odds ratio; CI, confidence interval.

**Note:** Adjusted by age, sex, SOFA, mechanical ventilation, renal replacement therapy, congestive heart failure, cardiac arrhythmias, valvular disease, peripheral vascular disease, pulmonary circulation disease, chronic pulmonary disease, hypothyroidism, hypertension, diabetes, renal failure, liver disease, tumor.

### 4.3 Association between the heart rate/temperature ratio and ICU mortality

**Fig. 2:**
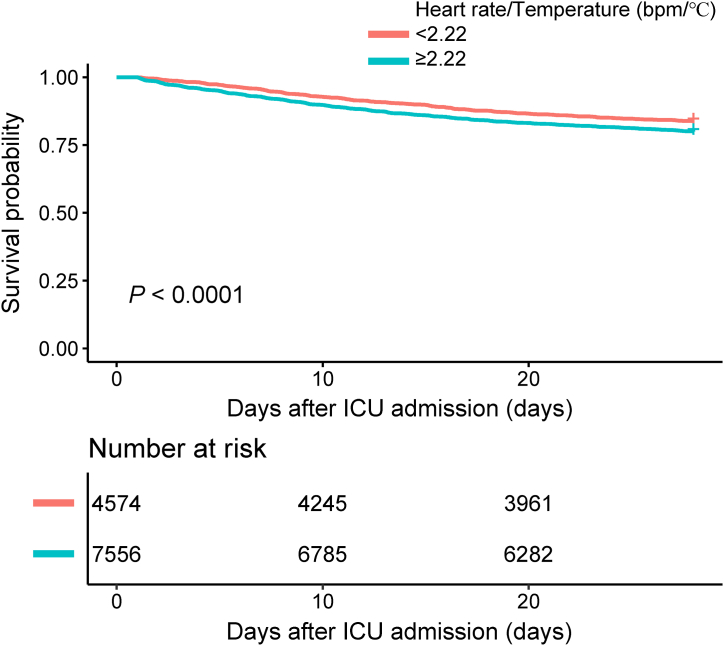
Survival curves for 28 days in different heart rate/temperature ratio groups.

**Fig. 3:**
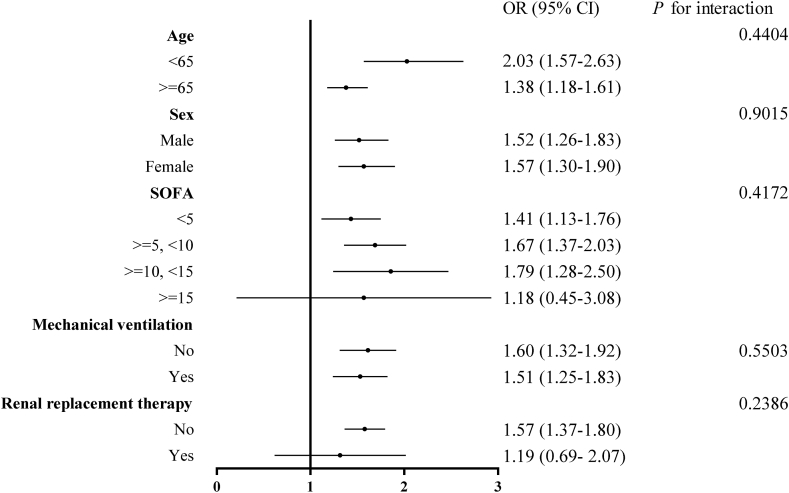
Forest plot of heart rate/temperature ratio on ICU mortality in prespecified and exploratory subgroups in each subgroup.

Multivariate logistic regression results demonstrated that the heart rate/temperature ratio was an independent risk factor for ICU mortality. In model II, a heart rate/temperature ratio greater than 2.22 was associated with a significant 55 % increase in ICU mortality, compared with less than 2.22 (OR = 1.55, 95%CI 1.35–1.77); similarly, an elevated heart rate/temperature ratio was positively associated with ICU mortality when it was used as a continuous variable (OR = 2.14, 95%CI 1.87–2.45) [Table 3]. In the stratified analysis, the effect of the heart rate/temperature ratio on ICU mortality remained consistent across all subgroup variables [Table 4 and Fig. 3]. Kaplan-Meier curves showed that the 28-day survival advantage was substantially higher in the heart rate/temperature ratio less than 2.22 group to the ratio greater than or equal to 2.22 group (log-rank test: *P* < 0.0001 [Fig. 2].

**Table 3:** Multivariable logistic regression analysis for ICU mortality.

Exposure	Crude (OR, 95 % CI, *P*-value)	Model I (OR, 95 % CI, *P*-value)	Model II (OR, 95 % CI, *P*-value)
Heart rate/Temperature (bpm/°C)			
<2.22	1.0	1.0	1.0
≥2.22	1.64 (1.44–1.86) <0.0001	1.63 (1.43–1.85) <0.0001	1.55 (1.35–1.77) <0.0001
Heart rate/Temperature (bpm/°C)	2.37 (2.08–2.70) <0.0001	2.33 (2.04–2.65) <0.0001	2.14 (1.87–2.45) <0.0001

**Abbreviations:** OR, odds ratio; CI, confidence interval.

**Note:** Model I adjust for: age, sex. Model II adjust for: age, sex, SOFA, mechanical ventilation, renal replacement therapy, congestive heart failure, cardiac arrhythmias, valvular disease, peripheral vascular disease, pulmonary circulation disease, chronic pulmonary disease, hypothyroidism, hypertension, diabetes, renal failure, liver disease, tumor.

**Table 4:** Effect size of heart rate/temperature ratio on ICU mortality in prespecified and exploratory subgroups in each subgroup.

Outcome: ICU mortality	Heart rate/Temperature <2.22	Heart rate/Temperature ≥2.22 (OR, 95 % CI)	*P*-value	Interaction *P*-value
Age (years)				0.4404
<65	1.0	2.03 (1.57–2.63)	<0.0001	
≥65	1.0	1.38 (1.18–1.61)	<0.0001	
Sex				0.9015
Male	1.0	1.52 (1.26–1.83)	<0.0001	
Female	1.0	1.57 (1.30–1.90)	<0.0001	
SOFA				0.4172
<5	1.0	1.41 (1.13–1.76)	0.0021	
≥5, <10	1.0	1.67 (1.37–2.03)	<0.0001	
≥10, <15	1.0	1.79 (1.28–2.50)	0.0007	
≥15	1.0	1.18 (0.45–3.08)	0.7395	
Mechanical ventilation				0.5503
No	1.0	1.60 (1.32–1.92)	<0.0001	
Yes	1.0	1.51 (1.25–1.83)	<0.0001	
Renal replacement therapy				0.2386
No	1.0	1.57 (1.37–1.80)	<0.0001	
Yes	1.0	1.19 (0.69–2.07)	0.5373	
Congestive heart failure				0.7101
No	1.0	1.57 (1.32–1.86)	<0.0001	
Yes	1.0	1.49 (1.21–1.84)	0.0002	
Cardiac arrhythmias				0.7969
No	1.0	1.57 (1.32–1.86)	<0.0001	
Yes	1.0	1.51 (1.22–1.86)	0.0001	
Valvular disease				0.5174
No	1.0	1.56 (1.35–1.80)	<0.0001	
Yes	1.0	1.47 (1.04–2.07)	0.0295	
Peripheral vascular disease				0.6896
No	1.0	1.55 (1.35–1.79)	<0.0001	
Yes	1.0	1.53 (1.07–2.20)	0.0197	
Pulmonary circulation disease				0.5184
No	1.0	1.58 (1.37–1.82)	<0.0001	
Yes	1.0	1.26 (0.81–1.96)	0.2970	
Chronic pulmonary disease				0.1629
No	1.0	1.65 (1.42–1.92)	<0.0001	
Yes	1.0	1.26 (0.96–1.66)	0.1004	
Hypothyroidism				0.7026
No	1.0	1.52 (1.32–1.75)	<0.0001	
Yes	1.0	1.77 (1.19–2.65)	0.0051	
Hypertension				0.7232
No	1.0	1.57 (1.30–1.91)	<0.0001	
Yes	1.0	1.51 (1.26–1.82)	<0.0001	
Diabetes				0.2860
No	1.0	1.60 (1.37–1.88)	<0.0001	
Yes	1.0	1.38 (1.08–1.77)	0.0109	
Renal failure				0.1688
No	1.0	1.61 (1.38–1.87)	<0.0001	
Yes	1.0	1.29 (0.97–1.72)	0.0800	
Liver disease				0.8876
No	1.0	1.53 (1.33–1.77)	<0.0001	
Yes	1.0	1.63 (1.15–2.32)	0.0059	
Tumor				0.0733
No	1.0	1.48 (1.28–1.71)	<0.0001	
Yes	1.0	1.97 (1.39–2.79)	0.0001	

**Abbreviations:** ICU, intensive care unit; SOFA, sequential organ failure assessment; OR, odds ratio; CI, confidence interval.

**Note:** Adjust by age, sex, SOFA, mechanical ventilation, renal replacement therapy, congestive heart failure, cardiac arrhythmias, valvular disease, peripheral vascular disease, pulmonary circulation disease, chronic pulmonary disease, hypothyroidism, hypertension, diabetes, renal failure, liver disease, and tumor except for the subgroup variable.

### 4.4 Sensitivity analysis

**Fig. 4:**
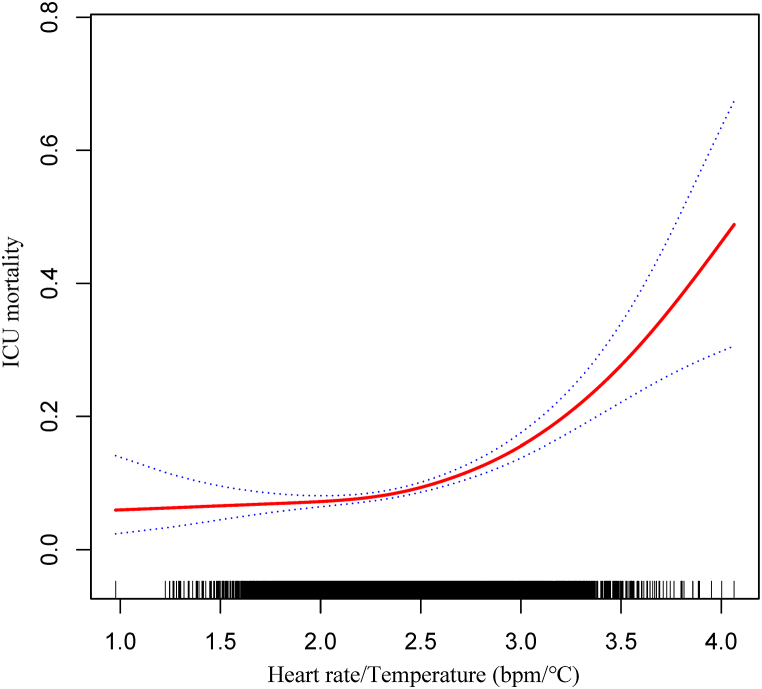
Sensitivity analysis of the relationship between heart rate/temperature ratio and ICU mortality. **Note:** adjusted by age, sex, SOFA, mechanical ventilation, renal replacement therapy, peripheral vascular disease, pulmonary circulation disease, chronic pulmonary disease, hypertension, diabetes, renal failure, liver disease, tumor.

In the sensitivity analysis, after excluding comorbidities that may directly affect heart rate, the results of multiple logistic regression similarly indicated that the heart rate/temperature ratio was an independent risk factor for ICU mortality, and the findings remained consistent [Table 5]. In Model II, the results of heart rate/temperature ratio, either as a categorical or continuous variable, suggested that a high heart rate/temperature ratio was positively associated with a poor prognosis in the ICU. The smooth curve fit plot showed an identical non-linear relationship between heart rate/temperature ratio and ICU mortality [Fig. 4]. In addition, the estimated E value of 2.47 implied that if the relative risk of both was ≥2.47 in the absence of immeasurable confounding factors related to the heart rate/temperature ratio as well as ICU mortality, then the results were obtained robustly.

**Table 5:** Sensitivity analysis of the association of heart rate/temperature ratio with ICU mortality.

Exposure	Crude (OR, 95 % CI, *P*-value)	Model I (OR, 95 % CI, *P*-value)	Model II (OR, 95 % CI, *P*-value)
Heart rate/Temperature (bpm/°C)			
<2.22	1.0	1.0	1.0
≥2.22	1.75 (1.41–2.17) <0.0001	1.75 (1.41–2.17) <0.0001	1.62 (1.30–2.02) <0.0001
Heart rate/Temperature (bpm/°C)	2.92 (2.37–3.59) <0.0001	2.89 (2.35–3.56) <0.0001	2.59 (2.09–3.23) <0.0001

**Abbreviations:** OR, odds ratio; CI, confidence interval.

**Note:** Model I adjust for: age, sex. Model II adjust for: age, sex, SOFA, mechanical ventilation, renal replacement therapy, peripheral vascular disease, pulmonary circulation disease, chronic pulmonary disease, hypertension, diabetes, renal failure, liver disease, tumor.

## 5 Discussion

In our study, we confirmed the relationship of heart rate/temperature ratio and ICU mortality in patients with sepsis, suggesting that a high heart rate/temperature ratio is strongly associated with adverse prognosis, which may enrich clinicians' prognostic assessment of patients with sepsis to proactively implement interventions.

Catecholamine release during sepsis results in sympathetic overstimulation, leading to tachycardia, impaired left ventricular diastolic filling, impaired coronary blood flow, and increased myocardial oxygen consumption. These factors can contribute to hemodynamic instability and unfavorable clinical outcomes [24]. The use of β-blockers may have protective effects against excessive levels of exogenous and endogenous catecholamines. Several studies have demonstrated the beneficial effects of beta blockers on the prognosis of patients with sepsis, particularly those with tachycardia [[12], [13], [14],^16^]. However, it is important to acknowledge that the interactions between the adrenergic pathways and the host's protective immunity are complex [25].

However, understanding the interactions between the adrenergic pathways and host-protective immunity is complicated [25]. Prolonged hypercatecholaminaemia directly hinders cellular immune activity by interacting with specific receptors [1]. For instance, the binding to β2-adrenergic receptors inhibits the production of cytokines and immunoglobulins by antigen-presenting cells [26]. It also obstructs the activation and function of immune cells by inhibiting the MAPK signaling pathway [27]. Catecholamines impede the production of various anti-inflammatory cytokines [28]. The hypothesis that β-blockers could mitigate the β-adrenergic effects in patients with sepsis was first explored by Berk et al., in 1969 [29]. They conducted a study in which dogs injected with endotoxins and subsequently administered propranolol had a higher survival rate and fewer congestive changes in the lungs and internal organs than dogs receiving endotoxins alone. Immunosuppression caused by hypercatecholamines differs from the later-developed immunosuppression in sepsis, which is characterized by an increased release of anti-inflammatory factors and apoptosis [30]. These factors have been established as indicators of poor prognosis in sepsis. This immune response contributes to an increase in the heart rate, which in turn leads to an elevation in the heart rate/temperature ratio. This may partially explain the association between the heart rate/temperature ratio and adverse outcomes in patients with sepsis.

Based on this understanding, early blockade of catecholamines through the use of β-blockers could potentially have a positive impact on the poor prognosis of sepsis. However, systematic reviews on this topic have concluded that the current evidence regarding the use of β-blockers in sepsis is still insufficient. The optimal timing, dosage, and therapeutic approach for their use have not been definitively established in studies [[31], [32], [33]].

A previous study conducted by Macchia et al. further confirmed the beneficial effects of long-term administration of-blockers on the short-term prognosis of critically ill patients with sepsis [34]. Additionally, a recent study by Guz et al. demonstrated that long-term β-blocker therapy was associated with reduced mortality in hospitalized patients with sepsis who showed absolute and relative tachycardia in a medical ward [16]. However, as mentioned earlier, the timing, dosage, and specific regimens for the use of β-blockers have yet to be further validated across these studies. Our study focused on assessing the predictive value of the heart rate/temperature ratio at the time of ICU admission for ICU prognosis. By using the heart rate/temperature ratio at the time of ICU admission, we aimed to minimize the confounding effects of medical therapies because ICU patients often undergo numerous interventions that could impact this ratio. The heart rate/temperature ratio reflects the organism's stress response to infection; a simultaneous increase in heart rate and body temperature will essentially maintain the ratio within a certain range; however, once an accelerated heart rate and hypothermia occur, the ratio will be greatly increased. Previous studies on poor prognoses related to hypothermia and tachycardia also support this interpretation. However, owing to the limited number of studies in this field, it is not yet sufficient to fully explain the predictive value of the heart rate/temperature ratio for ICU mortality. Nevertheless, an increasing number of studies have focused on the prognosis of sepsis to develop quicker and easier methods for its prediction. The initial determination of heart rate/temperature ratio, particularly when physiological and biochemical data are not readily available, can help enrich clinicians' assessment of patient outcomes. This direction of advancement holds promise for improving early detection and prediction of sepsis.

Despite the results of this study suggesting a robust positive correlation between heart rate/temperature ratio and ICU mortality, some limitations remain to be mentioned. First, the definition of the threshold for the heart rate/temperature ratio remained a concern, and we calculated the optimal inflection point through a two-segment linear regression model using threshold effects analysis. However, the value of this threshold in external data needs further investigation. Second, we did not consider the use of beta-blockers in the past and during medical treatment, which may affect the prognosis of patients with sepsis. However, previous studies have shown that the beneficial effects of beta-blockers are limited and influenced by numerous variables, and together with the calculation of E values, we found that unmeasured confounding factors could rarely modify the effect of heart rate/temperature ratio on outcome. Finally, causality was not available for interpreting the results of the association analysis, and external validity validation needs to be addressed in future prospective studies.

## 6 Conclusions

The heart rate/temperature ratio is a readily accessible and convenient indicator in the clinical environment. Elevated heart rate/temperature ratio, particularly those exceeding 2.22, are strongly associated with high ICU mortality rates among critically ill patients with sepsis. It is crucial for clinicians to remain attentive to these patients and proactively implement suitable interventions. Future studies should focus on confirming the precision of this threshold and investigating its suitability for diverse patient populations. This will enhance the differentiation between patients at high- and low-risk, allowing clinicians to administer more tailored and effective interventions.

## Data availability statement

The raw data itself is from a third-party dataset available from MIMIC-III, a freely accessible critical care database. Reproduction of their data is not permitted according to the Data Use Agreement of the database but access can be requested here: https://mimic.physionet.org/gettingstarted/access.

## Consent for publication

Not applicable.

## Funding information

This work was supported by the Key Project of the Affiliated Hospital of North Sichuan Medical College (2023ZD008).

## Ethics approval and consent to participate

The access of the database has been approved by the institutional review boards of both Beth Israel Deaconess Medical Center and Massachusetts Institute of Technology Affiliates (Record ID: 49780033).

## CRediT authorship contribution statement

**Zongbin Lin:** Writing – original draft, Data curation. **Shan Lin:** Writing – review & editing, Formal analysis, Conceptualization.

## Declaration of competing interest

The authors declare that they have no known competing financial interests or personal relationships that could have appeared to influence the work reported in this paper.

## Abbreviation

ICU: intensive care unit
SOFA: sequential organ failure assessment
IQR: interquartile range
CI: confidence interval; OR, odds ratio
